# Soluble Urokinase Receptor Levels Are Correlated with Focal Segmental Glomerulosclerosis Lesions in IgA Nephropathy: A Cohort Study from China

**DOI:** 10.1371/journal.pone.0138718

**Published:** 2015-09-18

**Authors:** Shui-Ming Guo, Min Han, Mei-Xue Chen, Yong Ning, Guang-Chang Pei, Yue-Qiang Li, Wei Dai, Shu-Wang Ge, Yuan-Jun Deng, Yan-Yan Guo, Xiao-Qing Li, Hermann Haller, Gang Xu, Song Rong

**Affiliations:** 1 Department of Nephrology, Division of Internal Medicine, Tongji Hospital, Tongji Medical College, Huazhong University of Science and Technology, Wuhan, Hubei, China; 2 Department of Nephrology, Hannover Medical School, Hannover, Germany; University of Utah School of Medicine, UNITED STATES

## Abstract

**Background:**

Soluble urokinase receptor (suPAR) may be involved in the pathological mechanisms of focal segmental glomerulosclerosis (FSGS) changes. However, it remains unclear whether suPAR is correlated with the FSGS-like lesions in IgA nephropathy (IgAN).

**Methods:**

We measured the plasma suPAR levels in 138 patients with IgAN, and then their clinical and pathological relationships were analyzed.

**Results:**

We found that the plasma suPAR levels were significantly correlated with age and renal function by both univariate and multivariate analysis in our IgAN patient cohort. Female had higher plasma suPAR levels and no significant correlation was observed between plasma suPAR levels and 24-h urine protein and highly sensitive C-reaction protein with multivariate analysis. In our cohort, sixty of these IgAN patients could be diagnosed with a type of FSGS lesions. The plasma suPAR levels were higher in the IgAN patients with FSGS lesions than in the IgAN patients without FSGS lesions by univariate (*P* < 0.0001) and multivariate (*P* < 0.001) analysis adjusting for other predictor variables, which might be helpful to differentiate the pathological changes with and without FSGS lesions. And the optimal cutoff value was 1806 pg/ml in this study. The plasma suPAR concentrations were also associated with the degree of tubular atrophy/interstitial fibrosis in both univariate and multivariate analysis. In multivariate analysis, the plasma suPAR levels were correlated with the percentage of crescents, not global sclerosis and arterial lesions.

**Conclusions:**

Our study suggested that the plasma suPAR levels were associated with age, gender, renal function, the degree of tubular atrophy/interstitial fibrosis and the percentage of crescent formation. The plasma suPAR might be a potential predictor for the presence of FSGS pathological lesions in Chinese patients with IgAN.

## Introduction

IgA nephropathy (IgAN) is the most common primary glomerular disease worldwide and defined by an IgA-dominant deposition within the glomerular mesangium [1]. Approximately 20%-50% of IgAN patients will reach end-stage renal disease by 20 years from the time of diagnosis [2]. The spectrum of clinical changes is wide, ranging from asymptomatic microscopic hematuria to rapidly progressive glomerulonephritis. In addition, the pathological changes vary from almost normal to crescentic glomerulonephritis or severe proliferative glomerulonephritis or to histopathological changes similar to focal segmental glomerulosclerosis (FSGS) [1, 3]. Previous studies have shown that several clinical factors, such as heavy proteinuria, uncontrolled hypertension, and renal dysfunction, can be used to predict the prognosis in IgA nephropathy. Both nephrologists and pathologists have attempted to discover the clinical relevance of these histopathological changes [1, 3–6].

It is well known that histopathological changes similar to FSGS frequently appear in IgAN [7, 8]. In 1997, Haas [9] directly incorporated FSGS as one of the classes into his histological subclassification of IgAN. Recently, Hill and his co-workers [10, 11] also provided evidence that the segmental sclerotic lesions in IgAN were very similar to those in primary FSGS. The authors found that the typical pathological changes of primary FSGS, including capsular adhesions and podocyte abnormality or loss, were extremely common in IgAN. Compared with patients without FSGS lesions, the IgAN patients with FSGS lesions are associated with a poorer renal outcome, and the FSGS lesions may therefore be useful as a potential predictor of outcome in IgAN patients [11, 12].

Recently, Wei *et al*. [13] found that soluble urokinase receptor (suPAR) may be related to FSGS. The authors showed that the circulating suPAR concentrations were remarkably elevated in the primary and recurrent FSGS patient cohorts and that the enhanced suPAR levels caused foot process effacement, proteinuria, and FSGS-like glomerular pathological changes through the podocyte β3-integrin signal pathway [13, 14]. However, it remains unclear whether circulating suPAR can be used as a specific diagnostic marker of FSGS. Huang *et al*. [15] showed that the plasma suPAR levels found in FSGS, although significantly elevated, were observed with other glomerular diseases, such as membranous nephropathy and minimal change disease. However, they did not distinguish between primary and secondary FSGS. Meijers *et al*. [16] also reported that suPAR did not differentiate the patients with idiopathic FSGS from those with non-FSGS chronic kidney disease in the patients with estimated glomerular filtration rate (eGFR) levels of more than 60, 45–60, 30–45 or < 30 ml/min per 1.73 m^2^. Moreover, Qin *et al*. [17] reported recently that suPAR may also be involve in the pathogenesis of lupus nephritis and that the suPAR plasma levels were significantly elevated in active lupus nephritis. However, the correlations between the suPAR levels and the FSGS-like lesions and disease activity in IgAN patients are still not clear.

In the present study, we measured the plasma suPAR levels in a cohort of patients with IgAN and then further analyzed their clinical and pathological relationships.

## Patients and Methods

### Patients

One hundred and thirty-eight adult (≥ 18 years) patients with IgAN at Tongji Hospital, Huazhong University of Science and Technology, from January 2013 to December 2013 were recruited into this study. All of the patients met the diagnostic criteria of IgAN and had complete clinical and pathological data. Patients with systemic lupus erythematosus, Henoch—Schönlein Purpura, chronic liver disease, diabetes mellitus or whose renal biopsy specimen contained less than 8 glomeruli were excluded.

This study complied with the Declaration of Helsinki Principles and was approved by the Ethics Committee of Tongji Hospital, Huazhong University of Science and Technology. Written informed consent was obtained from each patient.

### Blood sample collection

Plasma samples were collected from the patients on the day of renal biopsy and from 29 age- and gender-matched healthy donors as normal controls. The plasma samples were separated by centrifugation at 2000 g for 10 min and stored in aliquots at -80°C until use. During the study, repeated freezing and thawing of the samples was avoided.

### Plasma suPAR measurement

Plasma suPAR levels were measured using the Quantikine Human suPAR immunoassay (R&D Systems, Minneapolis, MN) according to the manufacturer’s protocol. Briefly, 100 μl assay diluent was added to each well of 96-well polystyrene microplates that were precoated with a mouse monoclonal antibody, followed by 50 μl plasma diluent, and incubated for 2 h at room temperature. Subsequently, the uPAR conjugate was added and incubated for 2 h at room temperature. Then, a substrate solution was added to each well and incubated for another 30 min at room temperature while protecting it from light. Finally, a stop solution was added to each well, and the absorbance was recorded using a Synergy 2 multimode reader (BioTek, Winooski, Vermont) at 450/570 nm. The suPAR level of each sample was calculated using Curve expert 1.4 (Curve Expert Software, Chattanooga, TN).

### Renal histology

The renal specimens were evaluated using direct immunofluorescence and light microscopy. All of the biopsy slides were reviewed by two pathologists blinded to the clinical data of the patients and then classified and graded according to the modified Columbia 2004 classification and the Oxford classification. Other pathologic changes in our cases were also described, including capsular adhesion, glomerular global sclerosis, glomerular crescents, and arterial lesions. Differences in the pathologists’ assessments were resolved by reviewing the biopsies again until a consensus was reached.

### Statistical analysis

The statistical analyses were performed using GraphPad Prism version 6 software (Graph software, San Diego, CA) and SPSS 17.0 software (SPSS, Chicago, IL). A comparison between the two groups was performed using the parametric t-test or the nonparametric Mann-Whitney test. Multiple comparisons among three groups were analyzed using a one-way ANOVA or the Kruskal-Wallis test. Correlations between the plasma suPAR levels and the pathological or clinical parameters were evaluated using Pearson’s correlation coefficient or the nonparametric Spearman’s correlation test. The optimal selection of the statistical method depended on the normality of the data distribution. All of the statistical analyses were two-tailed, and a *P* < 0.05 was considered to be statistically significant.

## Results

### Demographic and clinical characteristics of the patients in our cohort

We studied 138 plasma samples from patients with IgAN, whose demographic and clinical data are listed in Table 1. The median age of the patients was 36 years, ranging from 18 to 61 years. Of these, 53 were men and 85 were women. The mean arterial pressure (MAP) was 97 ± 14 mm Hg (23% of the patients had a blood pressure above the value of 140/90 mm Hg, and 15% were taking antihypertensive medication). Thirteen percent of the enrolled patients had previously received renin-angiotensin system (RAS) blockade therapy, and 4.3% had a known previous tonsillectomy. Of the 138 patients, 133 had microscopic hematuria (96.4%). The mean serum albumin was 38.3 ± 5.4 g/l. The patients’ median serum creatinine (Scr), eGFR, and 24-h urine protein were 75.0 (IQR 60.8–108.0) μmol/l, 96.6 (IQR 71.1–117.0) ml/min per 1.73 m^2^, and 0.86 (IQR 0.45–1.44) g/d, respectively, at the time of biopsy. The median of the highly sensitive C-reactive protein (hsCRP) was 0.5 (IQR 0.2–1.3) mg/l.

**Table 1:** The demographic and clinical parameters of the patients with IgA nephropathy.

Parameters	Results
Age (years; median, range)	36,18–61
Gender (female/male)	85/53
MAP (mm Hg; mean ± s.d.)	97 ± 14
Taking antihypertensive medication, n (%)	21 (15%)
Treated with RAS blockade, n (%)	18 (13%)
Known previous tonsillectomy, n (%)	6 (4.3%)
Microscopic hematuria, n (%)	96.4
Albumin (g/l; mean ± s.d.)	38.3 ± 5.4
Serum creatinine (μmol/l; median, IQR)	75.0, 60.8–108.0
eGFR (ml/min per 1.73 m^2^; median, IQR)	96.6, 71.1–117.0
24-h urine protein (g/d; median, IQR)	0.86, 0.45–1.44
hsCRP (mg/l; median, IQR)	0.5, 0.2–1.3

Values are expressed as mean ± standard deviation, median (25th percentile-75th percentile), or number (percentage). MAP, mean arterial pressure; RAS, renin-angiotensin system; eGFR, estimated glomerular filtration rate; hsCRP, highly sensitive C-reaction protein; IQR, interquartile range. eGFR was calculated with CKD-EPI equation. 24-h urine protein was missing in 13% of cases.

### The associations between the plasma suPAR levels and the clinical data of the patients with IgAN:univariate linear regression analysis

**Fig 1:**
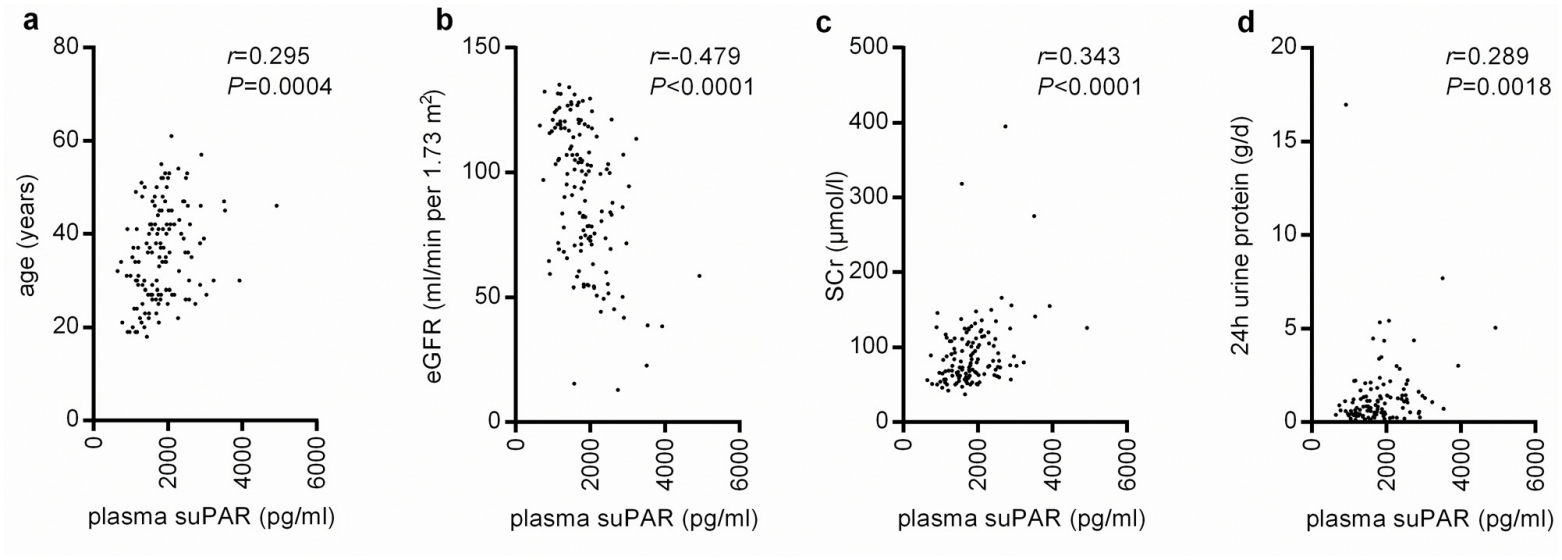
The correlations between the plasma soluble urokinase receptor (suPAR) with age (a), eGFR (b), SCr (c) and 24-h urine protein (d) in univariate analysis. eGFR, estimated glomerular filtration rate; SCr, serum creatinine.

We assessed the associations between the patients’ plasma suPAR levels and their clinical parameters. Age was directly correlated with the plasma suPAR concentrations in the overall patient cohort (*r* = 0.295, *P* = 0.0004; Fig 1a). Previous studies have revealed that the circulating suPAR concentration has an inverse correlation with the renal function [16, 18]. In the current investigation, the plasma suPAR levels in the patients with IgAN had a negatively significant correlation with the eGFR (*r* = -0.479, *P* < 0.0001; Fig 1b). Moreover, the plasma suPAR levels were also directly correlated with the Scr and 24-h urine protein (*r* = 0.343, *P* < 0.0001; *r* = 0.289, *P* = 0.0018, respectively; Fig 1c and 1d). A marked association was also observed with the red blood cells (*P* = 0.004), hemoglobin (*P* = 0.02), blood urea nitrogen (*P* = 0.001), and highly sensitive C-reactive protein (*P* = 0.008). The details are listed in Table 2.

**Table 2:** Correlations between the levels of plasma suPAR and other clinical parameters in the patients with IgA nephropathy in univariate analysis.

Clinical parameter	*r*-value	*P*-value
Red blood cell (× 10^12^/l)	-0.248	0.004
Hemoglobin (g/l)	-0.2	0.02
White blood cell (× 10^9^/l)	0.045	0.602
Platelet (× 10^9^/l)	-0.001	0.993
BUN (mmol/l)	0.276	0.001
Uric acid (μmol/l)	0.056	0.519
urine erythrocyte (/μl)	-0.031	0.723
leucocyturia (/μl)	-0.017	0.848
hsCRP (mg/l)	0.227	0.008

BUN, blood urea nitrogen; hsCRP, highly sensitive C-reaction protein.

### FSGS lesions and the Oxford classification in the cohort

**Fig 2:**
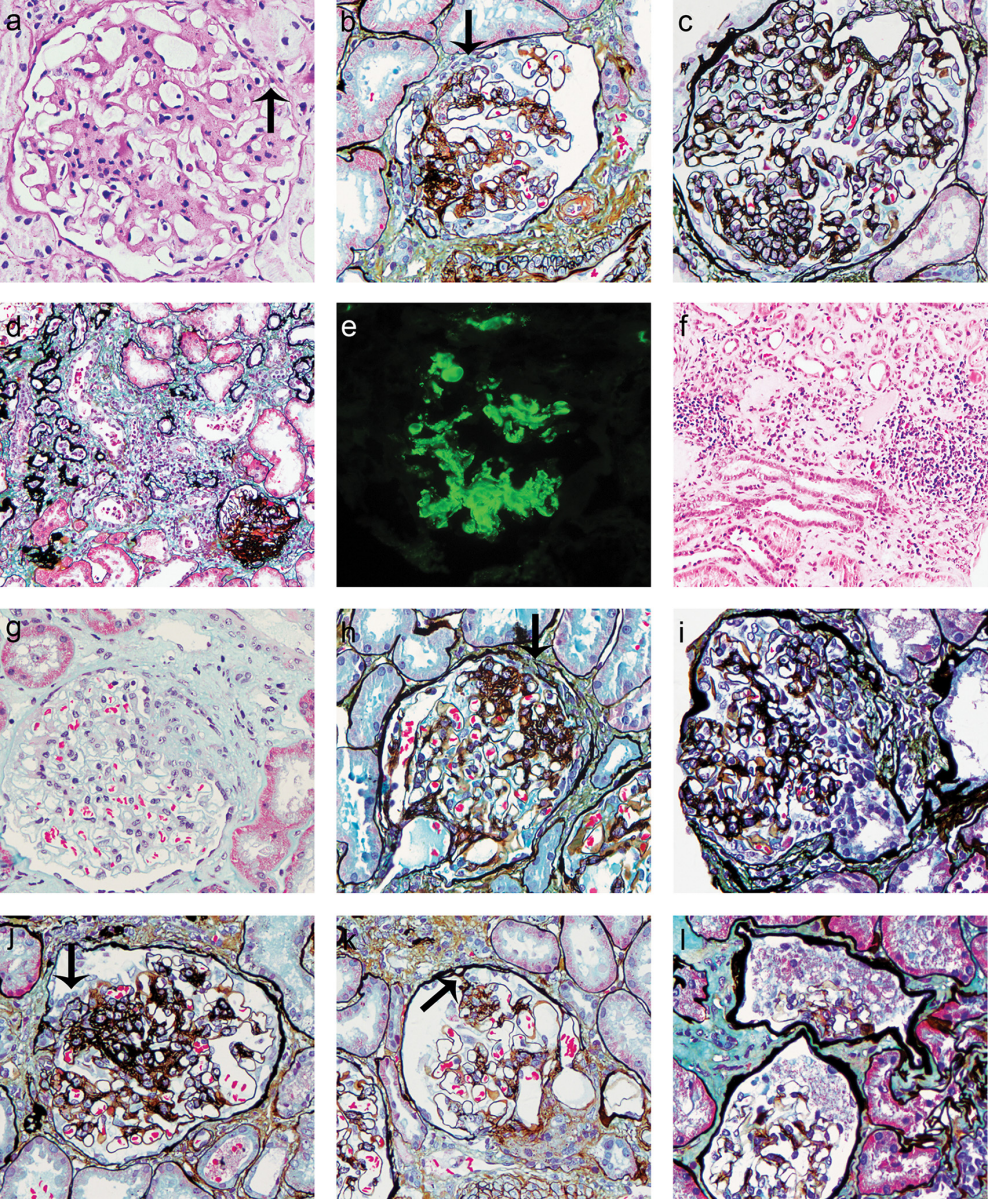
FSGS lesions in IgA nephropathy. (a) Oxford criterion—mesangial hypercellularity. Multiple mesangial areas show more than four mesangial cells. Capsular adhesions are also observed (arrow). Periodic acid-schiff (PAS), original magnification × 400. (b) Oxford criterion—segmental glomerulosclerosis. The segmental scar shows significant epithelial proliferation and capsular adhesion (arrow), thus it also defined as a type of FSGS lesion. Periodic acid-silver methenamine (PASM), original magnification × 400. (c) Oxford criterion—endocapillary hypercellularity. Many of the lobules show endothelial cell proliferation in the capillary lumens. PASM, original magnification × 400. (d) Oxford criterion—tubular atrophy/interstitial fibrosis. They accompany the serious sclerotic lesions of a glomerulus. PASM, original magnification × 200. (e) Immunofluorescence for IgA. Segmental IgA-dominant deposition within the glomerular mesangium. original magnification × 400. (f) Interstitial infiltration. Local significant interstitial inflammatory cell infiltration. Hematoxylin and eosin (HE), original magnification × 200. (g) Perihilar variant of an FSGS lesion. Sclerosis appears at the vascular pole. Perihilar sclerosis and/or hyalinosis were identified in more than 50% of the glomeruli with segmental lesions. Masson trichrome, original magnification × 400. (h) Tip lesion. Segmental sclerosis lesions appear at the tubular pole with adhesions of epithelial cells with parietal cells at the tubular neck (arrow). PASM, original magnification × 400. (i) Cellular lesions of FSGS. Segmental endocapillary hypercellularity with significant epithelial hyperplasia. PASM, original magnification × 400. (j) Not otherwise specified (NOS) FSGS lesions. Segmental scar with capsular adhesion and hyperplasia of epithelium (arrow). PASM, original magnification × 400. (k) Capsular adhesion. The glomerulus presents adherence to the capsule (arrow) with an epithelial cell proliferation reaction, consistent with the NOS variant of FSGS. PASM, original magnification × 400. (l) Pseudocrescent in Bowman’s space. Proliferous epithelial cells fill most of the capsule space, without blocking it. PASM, original magnification × 400.

To classify our cases of IgAN more stringently in terms of their FSGS lesions, we used a modified version of the Columbia 2004 classification of FSGS, as Hill *et al*. [11] have reported. For the definitions of the tip, perihilar and collapsing glomerulopathy variants of FSGS, we directly adopted the Columbia criteria. Nevertheless, to distinguish them from a simple segmental scar, the classification requires that the not otherwise specified (NOS) FSGS, at a minimum, should also have either overlying epithelial proliferation or hyalinosis lesions. For the diagnosis of cellular FSGS, the presence of hyperplastic epithelium overlying the areas of endocapillary cellularity was required to differentiate possible endocapillary hypercellylarity from other causes (Fig 2).

According to the modified classification, we found 60 patients in our IgAN cohort with glomerular lesions that were definable as FSGS lesions, including 43 cases qualifying as NOS variants, 9 perihilar variant cases, 2 cases diagnosable as cellular variants, 6 tip lesions, and no cases with collapsing glomerulopathy. Four of the Oxford criteria were also met by our cohort. There were 91 patients with mesangial hypercellularity (M) in > 50% of the glomeruli, and in 52 (57%) of those patients, the mesangial hypercellularity was accompanied by FSGS lesions. Segmental glomerulosclerosis (S) as defined in the Oxford classification occurred in 100 (72.5%) of the patients, which included all of the cases diagnosed as FSGS. Endocapillary hypercellularity (E) was found in 23 patients, and 17 (73.9%) cases can be classified as FSGS. According to the classification of tubular atrophy/interstitial fibrosis (T), the numbers of the T0, T1, and T2 lesions were 72 (52%), 43 (31%), and 23 (17%) cases, respectively. There were 18 (25%) samples interpretable as FSGS in T0 lesions, 26 (60%) cases in T1 lesions, and 16 (70%) cases in T2 lesions.

### The plasma suPAR levels in the patients with IgAN in terms of the FSGS lesions:univariate linear regression analysis

**Fig 3:**
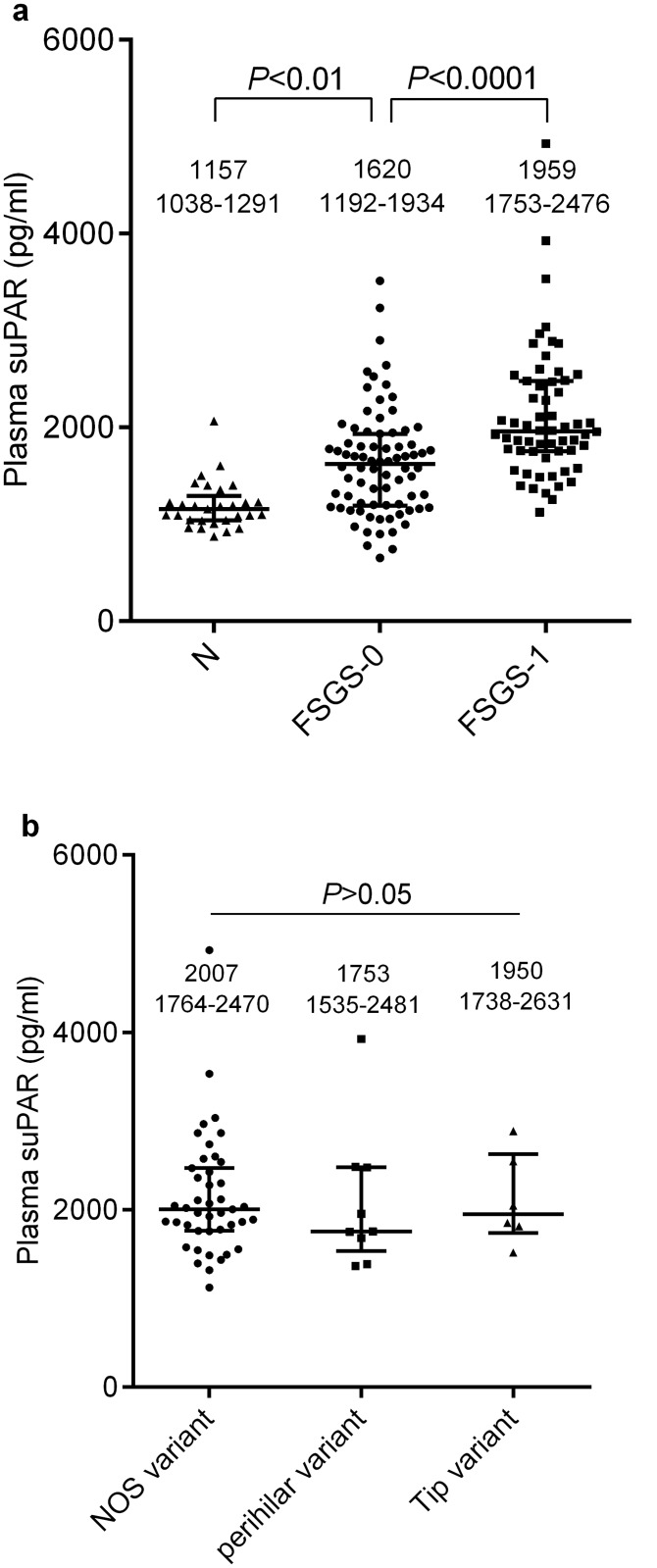
(a) Plasma soluble urokinase receptor (suPAR) levels among the IgAN patients with FSGS lesions, without FSGS lesions, and normal subjects. FSGS-0: patients without FSGS; FSGS-1: patients with FSGS lesions; N: normal subjects. (b) The plasma soluble urokinase receptor (suPAR) levels in the IgAN patients with different FSGS pathological variants. NOS, not otherwise specified.

As shown in Fig 3a, the plasma suPAR levels of the IgAN patients with FSGS lesions (1959, IQR 1753–2476 pg/ml) were significantly higher than those of the patients without FSGS lesions (1620, IQR 1192–1934 pg/ml, *P* < 0.0001) and the normal controls (1157, IQR 1038–1291 pg/ml, *P* < 0.0001); there was also a significant difference between the latter two groups (*P* < 0.01). We also compared the plasma suPAR levels among the different types of the FSGS lesions. As shown in Fig 3b, the plasma levels among the NOS, perihilar, and tip variants (2007, IQR 1764–2470 pg/ml; 1753, IQR 1535–2481 pg/ml; 1950, IQR 1738–2631 pg/ml, respectively) did not reach statistical significance (*P* > 0.05). Because there were only two cases with cellular variants, a statistical analysis was not performed for them; their plasma suPAR levels were 1253 and 1926 pg/ml.

### The correlations between plasma suPAR levels and FSGS lesions and clinical parameters: multivariate linear regression analysis

Next, we assessed the associations between the FSGS lesions and relevant clinical data and their plasma suPAR concentrations by a multiple regression analysis. Because the eGFR is calculated by age, gender and serum creatinine which are all the potential impact elements of plasma suPAR levels, we adopted serum creatinine instead of eGFR as the renal function index [18]. As shown in Table 3, the multivariate analysis model also included age, gender, mean arterial pressure, FSGS lesions, 24-h urine protein, highly sensitive C-reaction protein and disease duration time. The results showed that FSGS lesions in IgAN patients were still significantly correlated with the plasma suPAR levels after controlling other parameters (*P* < 0.001). Age and serum creatinine still had significantly associations with the plasma suPAR levels (*P* = 0.002 and *P* < 0.001, respectively). The suPAR values were higher in female (*P* = 0.002). In multivariate analysis, no significant independent association was observed between the suPAR levels and highly sensitive C-reaction protein and 24-h urine protein. The plasma suPAR levels in different FSGS pathology subtypes were still similar (*P* > 0.05) after adjusting for the above predictor variables (data not shown).

**Table 3:** Multiple regression analysis of associations between plasma suPAR levels and FSGS lesions and clinical parameters in IgAN patients^a^.

	β	s.e.m.	*P*-value
Age (years)	17.7	5.6	0.002
Gender (female)	374.7	118.4	0.002
MAP (mm Hg)	3.8	4.1	0.361
FSGS lesions	437.7	108.5	<0.001
Serum creatinine (μmol/l)	5.2	1.4	<0.001
24-h urine protein (g/d)	12.2	29.8	0.684
hsCRP (mg/l)	24.0	13.9	0.088
Disease duration time (month)	-2.9	1.9	0.120

^a^Multivariate model: multivariate with FSFG lesions plus age, gender, mean arterial pressure, serum creatinine, 24-h urine protein, highly sensitive C-reaction protein and disease duration time.

MAP, mean arterial pressure; FSGS lesions, focal segmental glomerulosclerosis lesions; hsCRP, highly sensitive C-reaction protein.

### The diagnostic value of the suPAR: differentiating the IgAN patients with FSGS lesions from those without FSGS lesions

Previous studies have reported that patients with FSGS lesions had significantly poorer renal outcomes than patients without FSGS lesions in their IgAN cohort [11, 12]. In our cohort, the plasma suPAR levels of the patients with FSGS lesion were significantly higher than those of the patients without FSGS lesions. Moreover, an independent correlation exists between them, as described above. Thus, we performed a receiver operating characteristic (ROC) curve analysis on the two subgroups to analyze the predictive value of suPAR for the presence of FSGS lesions in patients with IgAN. The optimal cutoff value was 1806 pg/ml. The sensitivity and specificity were 0.683 and 0.718, respectively. The positive likelihood ratio was 2.42, and the negative likelihood ratio was 0.442. The area under the ROC curve for this patient cohort was 0.731 ± 0.042 (95% confidence interval (CI): 0.647–0.814, *P* < 0.0001). These data suggest that the plasma suPAR level is a valuable factor for differentiating the IgAN patients with FSGS lesions from those without FSGS lesions.

### The plasma suPAR levels in the patients with IgAN in terms of the Oxford classification: univariate and multivariate linear regression analysis

We also analyzed the relationship between the plasma suPAR levels in our cohort and the four Oxford criteria for mesangial hypercellularity, endocapillary hypercellularity, segmental glomerulosclerosis and tubular atrophy/interstitial fibrosis, as shown in Table 4. With univariate analysis, the plasma suPAR concentrations in the patients with mesangial hypercellularity (1853, IQR 1578–2312 pg/ml) were substantially higher than those of the patients without mesangial hypercellularity (1495, IQR 1169–1859 pg/ml, *P* = 0.0001). The plasma suPAR values were also significantly higher in the patients with endocapillary hypercellularity (2007, IQR 1753–2539 pg/ml) compared with those without endocapillary hypercellularity (1734, IQR 1374–2036 pg/ml, *P* = 0.005). The patients with segmental glomerulosclerosis as defined in the Oxford classification (1844, IQR 1489–2283 pg/ml) showed higher levels than those without such pathological variations (1655, IQR 1167–1804 pg/ml, *P* = 0.002). The patients with tubular atrophy/interstitial fibrosis of ≤ 25% had lower plasma suPAR concentrations (1579, IQR 1197–1847 pg/ml) than those with tubular atrophy/interstitial fibrosis 26%-50% (1931, IQR 1577–2285 pg/ml, *P* < 0.01) and > 50% (2109, IQR 1826–2738 pg/ml, *P* < 0.0001), and there was no significant difference between the latter two groups. For eliminating the influence of some confounding factors on the univariate analysis results, a multivariate analysis was performed. The four Oxford criteria, age, gender, MAP, serum creatinine, 24-h urine protein and disease duration time were included in this model. As listed in Table 4, the plasma suPAR levels still were significantly correlated with the degree of tubular atrophy/interstitial fibrosis after adjustment for other predictor variables (*P* = 0.041). We did not find the other three pathological changes had independent correlation with the plasma suPAR levels in the multivariate analysis model. The significant associations between plasma suPAR levels and age, gender and renal function were consistent with the above-described results.

**Table 4:** Correlations between plasma suPAR levels and the Oxford classification in IgAN patients: univariate and multivariate linear regression analysis.

	Univariate Linear Regression	Multivariate Linear Regression^a^
	Level of suPAR (pg/ml; median, IQR)	β (s.e.m.)
Mesangial hypercellularity		85 (136)
Score ≤ 0.5	1495, 1169–1859	
Score ≥ 0.5	1853, 1578–2312	
	*P* = 0.0001	*P* = 0.532
Endocapillary hypercellularity		186 (152)
Absent	1734, 1374–2036	
Present	2007, 1753–2539	
	*P* = 0.005	*P* = 0.223
Segmental glomerulosclerosis^b^		180 (136)
Absent	1655, 1167–1804	
Present	1844, 1489–2283	
	*P* = 0.002	*P* = 0.189
Tubular atrophy/interstitial fibrosis		194 (94)
0–25%	1579, 1197–1847	
26–50%	1931, 1577–2285	
>50%	2109, 1826–2738	
	*P*<0.0001	*P* = 0.041

^a^Multivariate model: multivariate with the four pathologic features (mesangial hypercellularity, endocapillary hypercellularity, Segmental glomerulosclerosis, tubular atrophy/interstitial fibrosis) plus age, gender, mean arterial pressure, serum creatinine, 24-h urine protein and disease duration time.

^b^Segmental glomerulosclerosis is defined as “any amount of the tuft involved in sclerosis, but not involving the whole tuft or the present of an adhesion”, as described in the Oxford classification, and is different from FSGS lesions in IgAN.

### The associations between the plasma suPAR levels and the other pathological data of the patients with IgAN

Then, we evaluated the associations between the plasma suPAR levels and the crescent formations, global sclerosis and arterial lesions. The plasma suPAR levels were not significantly correlated with the percentage of the crescent formation in our IgAN cohort by univariate analysis (*r* = 0.131, *P* = 0.126); however, there was a remarkable correlation between them after adjusting for other variables by a multivariate analysis (*P* = 0.006). Although the plasma suPAR levels were significantly associated with global sclerosis (*P* < 0.0001) and arterial lesions (*P* < 0.0001) with univariate analysis, the differences were not observed in multivariate analysis (*P* = 0.520; *P* = 0.076, respectively). The details are shown in Table 5.

**Table 5:** Associations between plasma suPAR levels and other pathological changes in IgAN patients: univatiate and multivariate linear regression analysis.

	Univariate Linear Regression		Multivariate Linear Regression^a^	
	*r*	*P*-value	β (s.e.m.)	*P*-value
Crescent number (%)	0.131	0.126	16 (6)	0.006
Global sclerosis number (%)	0.344	<0.0001	4 (6)	0.520
Arterial lesions	0.356	<0.0001	176 (99)	0.076

^a^Multivariate model: multivariate with the three pathologic features (crescent, global sclerosis and artery lesions) plus age, gender, mean arterial pressure, serum creatinine, 24-h urine protein and disease duration time.

### The association between the FSGS lesions and renal outcomes

Ninety patients were followed up in our cohort, including 41 cases with FSGS lesions and 49 without FSGS lesions. The average length of the follow-up was 16 months. One patient required dialysis treatment and the Scr values of one patient in the FSGS lesions subgroup doubled. The plasma suPAR concentrations in these two patients were 2737.85 pg/ml and 2108.6 pg/ml, respectively, and both of their Oxford classification were M1E0S1T2. None of the patients in non-FSGS lesions subgroup had poor renal outcomes.

## Discussion

As Wada *et al*. [18] has also reported, we showed that the plasma suPAR levels were significantly correlated with age by either univariate or multivariate analysis. Females had higher plasma suPAR levels in our patient cohort by multivariate analysis, which also was consist with the result reported by Meijers *et al* [16]. Similar to previous investigations, our study also demonstrated a negative correlation between the suPAR and the renal function indictors eGFR and SCr in our IgAN patient cohort. One reason for the inverse relationship is that the loss of glomerular filtration reduces the urinary excretion of suPAR, as a circulating protein ranging from 20 to 50 KD [16, 19] and results in rising suPAR concentrations. However, Franco Palacios *et al*. [20] recently reported that the urine suPAR rather than the serum suPAR before transplantation was significantly higher in cases with recurrent FSGS compared with other end-stage renal disease patients with diabetic nephropathy, membranous nephropathy, IgAN, or autosomal dominant polycystic kidney disease and normal subjects. Huang *et al*. [21] also showed that the urine suPAR levels in patients with primary FSGS were significantly higher than in patients with minimal change disease, membranous nephropathy, secondary FSGS and normal subjects; in addition, there was no association between the urine suPAR level and the eGFR in any of the glomerular disease subgroups. These studies suggested that the decline in the renal function might not be the only cause of the accumulation of plasma suPAR in patients with kidney disease. We also suspected that the accumulation of plasma suPAR might be the common result of its overproduction during kidney disease and the loss of glomerular filtration. Although the plasma suPAR level was associated with urinary protein excretion and hsCRP by univariate analysis, no significant correlation was found after adjustment for the differences in age, gender and renal function. The results showed that urine protein and inflammation were not the major independent relevant factors of plasma suPAR levels in IgAN patients.

Our study showed the FSGS lesions were present in IgAN based on the modified version of the Columbia 2004 classification of primary FSGS. In our IgAN cohort, we found 60 patients (43%) who could be diagnosed with a type of FSGS lesions, including 43 with NOS variants, 9 with perihilar variants, 2 with cellular variants, 6 with tip variants and none with a collapsing variant, which was a lower proportion than the 79% previously reported by Hill *et al*. [11] in a cohort with more advanced disease. We also classified all patients using the Oxford classification and found that 65.9% of the patients had mesangial hypercellularity, 72.5% had segmental glomerulosclerosis, and 16.7% had endocapillary hypercellularity. In regard to tubular atrophy/interstitial fibrosis, 52% of the patients were graded as T0, 31% as T1, and 17% as T2. We found that most of the pathological variations in the Oxford criteria were accompanied by FSGS lesions, as similarly reported by Hill *et al*.[11].

For the association between the plasma suPAR levels and the presence of FSGS lesions in IgAN, our study indicated that the plasma suPAR levels were significantly higher in the patients with FSGS lesions than in the patients without FSGS lesions by univariate (*P* < 0.0001)and multivariate (*P* < 0.001) analysis adjusting for other predictor variables, such as age, gender, MAP and renal function etc, which was consistent with data reported by Wei *et al*. [13, 14] in two studies on primary FSGS. We then investigated whether the plasma suPAR values were accurate for calculating the presence of FSGS lesion in IgAN using an ROC curve analysis. We observed that the suPAR could effectively differentiate the IgAN patients with FSGS lesions from those without lesions. The optimal cutoff value was 1806 pg/ml, and the sensitivity and specificity were 0.683 and 0.718, respectively. The cutoff value was remarkably lower than the 3000 pg/ml used as a cutoff value for diagnosing primary FSGS in Wei’s studies, which may be the result of the different populations, the types of disease, or test instruments. The findings suggest that plasma suPAR levels could independently predict the presence of FSGS lesions in IgAN patients. FSGS has various pathological phenotypes, which may reflect different pathological mechanisms. In our study, the plasma suPAR levels increased in the order of perihilar, tip, and NOS variants, and there was no significant difference between them by univariate and multivariate analysis. Two cases with cellular variants had relatively low plasma suPAR concentrations of 1253 and 1926 pg/ml. These results were not consistent with the finding Huang *et al*. [15] reported in a study on primary FSGS that showed that patients with cellular variants had higher plasma suPAR levels compared with patients with the tip and NOS variants. The limited number of patients may explain this discrepancy. A larger population is required for clarifying the differences in the etiology and pathogenesis among the different morphologic subtypes of FSGS.

Then, we also analyzed the relationships between plasma suPAR level and pathological factors in the Oxford classification. We found that the presence of mesangial hypercellularity, endocapillary hypercellularity, or segmental glomerulosclerosis, according to Oxford classification, was significantly associated with higher plasma suPAR concentrations by univariate analysis, but in multivariate analysis these pathological changes were not correlated with plasma suPAR levels except for the degree of tubular atrophy/interstitial fibrosis. Recently, many studies have tried to verify the results from the Oxford classification by different populations or sample sizes [22–27]. The prognostic values of mesangial hypercellularity, endocapillary hypercellularity, and segmental glomerulosclerosis were inconsistent among the study. However, tubular atrophy/interstitial fibrosis was considered consistently to be a predictor factor in all studies. In addition, FSGS lesions in IgAN patients also were correlated with poor outcomes [11, 12]. Moreover, we also found there was a significant association between the plasma suPAR levels and the percentage of glomeruli crescent formation. Accordingly, we speculated that the plasma suPAR level might have a potent prognostic ability for renal outcomes in IgAN, as Qin *et al*. [17] reported on the role of plasma suPAR responses to disease activity in lupus nephritis in a large Chinese patient cohort.

As mentioned before, we found FSGS lesions in IgAN patients were significantly correlated with the plasma suPAR levels, but no association was found between the plasma suPAR levels and segmental glomerulosclerosis defined in the Oxford classification in multivariate analysis. The difference could be explained by the following aspects. Firstly, the segmental glomerulosclerosis described in the Oxford classification includes capsular adhesion, which presented at a high frequency in our cohort compared with the FSGS lesions (72.5% versus 43.5%). And capsular adhesion alone was not significantly correlated with the plasma suPAR levels in a multivariate analysis (*P* = 0.638). Secondly, the FSGS lesions defined in our study excluded simple and inactive segmental scar without epithelial proliferation, according to the modified Columbia criteria.

There were many exciting studies about suPAR in renal disease recently, although some studies did not support even against the role of suPAR in chronic kidney disease, especially primary FSGS [28, 29], based on current defect of technology and the influence of renal function on it. Huang *et al*. reported the plasma suPAR levels of patient with primary FSGS were still higher than those of patients with minimal change disease (*P* = 0.01) or membranous nephropathy (*P* = 0.003) after adjusting for renal function [30]. Plasma suPAR levels were correlated with a higher risk of new-onset cardiovascular events form a European study [31] and active lupus nephritis from a China cohort study [17]. In type 1 diabetes patients, plasma suPAR values were higher and correlated with diabetic kidney damage [32]. The shifting of binding site of suPAR from acid sphingomyelinase-like phosphodiesterase 3b to β3 integrin induced different podocyte injury phenotype—an apoptotic diabetic kidney disease or migratory FSGS-like phenotype [33]. The role of suPAR in the pathogenesis of FSGS lesions in IgAN might include the following aspects. First, the existence of multiple suPAR variations caused podocyte injury in IgAN patients, such as heavily glycosylated suPAR, whose role may be more apparent. Second, there may be synergy factors of suPAR in IgAN patients, for instance, aggregated IgA [34], immune complex or complement component. Third, many studies have demonstrated that mesangial cell-derived factors induced by aberrantly glycosylated IgA1, such as platelet-activating factor and tumor necrosis factor-α, inhibited nephrin expression and adhesive capacity of podocytes [34–36]. Shushakova *et al*. [37] recently reported that urokinase could potentiate the C5a/C5aR-related release of inflammatory factors of mesangial cells in human mesangial cells. Accordingly, we hypothesized that suPAR-inducing podocyte injury in IgAN might result from indirectly influence on mesangial cells. Fourth, the direct effect of suPAR on podocytes by β3 integrin pathway may also occur in IgAN [13].

Several issues may affect the results obtained in this study. First, although our study suggests that the plasma suPAR level may be an independent biomarker for FSGS lesions in IgAN, we cannot explain accurately how suPAR directly or indirectly promotes the pathogenesis and development of FSGS lesions in IgAN, which will require further study. Second, the sample size of the study was relatively small. A multicenter study should also be undertaken to reduce the occurrence of population and test biases. Third, the stability and specificity of the commercial ELISA kits should be improved.

In conclusion, the results of this study suggested that the plasma suPAR levels were associated with age, gender, renal function, the degree of tubular atrophy/interstitial fibrosis and the percentage of crescent formation. The plasma suPAR might be a potential predictor for the presence of FSGS pathological lesions in Chinese patients with IgAN.
